# Maintaining homeostasis by controlled alternatives for energy distribution in plant cells under changing conditions of supply and demand

**DOI:** 10.1007/s11120-018-0583-z

**Published:** 2018-09-10

**Authors:** Renate Scheibe

**Affiliations:** Department of Plant Physiology, Faculty of Biology and Chemistry, University of Osnabrueck, 49069 Osnabrueck, Germany

**Keywords:** Biomass production, Energy metabolism, GAPDH, Glycolysis, Malate valves, Moonlighting, Nitrogen nutrition, Photosynthesis

## Abstract

Plants depend on light energy for the generation of ATP and reductant as well as on supply of nutrients (inorganic C, N, and S compounds) to successfully produce biomass. Any excess of reducing power or lack of electron acceptors can lead to the formation of reactive oxygen species (ROS). Multiple systems are operating to avoid imbalances and subsequent oxidative stress by efficiently scavenging any formed ROS. Plants can sense an upcoming imbalance and correspondingly adapt to changed conditions not only by an increase of ROS scavengers, but also by using excess incoming light energy productively for assimilatory processes in actively metabolizing cells of growing leaves. CO_2_ assimilation in chloroplasts is controlled by various redox-regulated enzymes; their activation state is strictly linked to metabolism due to the effects of small molecules on their actual activation state. Shuttle systems for indirect transfer of reducing equivalents and ATP specifically distribute the energy fluxes between compartments for optimal biomass production. Integration of metabolic and redox signals involves the cytosolic enzyme glyceraldehyde-3-P dehydrogenase (GapC) and some of its many moonlighting functions. Its redox- and metabolite-dependent interactions with the mitochondrial outer membrane, the cytoskeleton, and its occurrence in the nucleus are examples of these additional functions. Induction of the genes required to achieve an optimal response suitable for the respective conditions allows for growth when plants are exposed to different light intensities and nutrient conditions with varying rates of energy input and different assimilatory pathways for its consumption are the required in the long term. A plant-specific respiratory pathway, the alternative oxidase (AOX), functions as a site to convert excess electrons into heat. For acclimation, any imbalance is sensed and elicits signal transduction to induce the required genes. Examples for regulated steps in this sequence of events are given in this review. Continuous adjustment under natural conditions allows for adaptive responses. In contrast, sudden light stress, as employed when analyzing stress responses in lab experiments, frequently results in cell destruction. Knowledge of all the flexible regulatory mechanisms, their responsiveness, and their interdependencies is needed when plant growth is to be engineered to optimize biomass and production of any desired molecules.

## Introduction

Plants as sessile organisms which depend on light as the primary energy source cannot easily escape stressful conditions. Therefore, their energy metabolism requires permanent adjustment to avoid imbalances and formation of harmful radicals. Particularly the ATP/ADP and NAD(P)H/NAD(P)^+^ ratios need to be balanced in each cellular compartment as well as the ATP/NADPH ratios therein. In each compartment, specific isoenzymes of basic metabolism provide reductants and ATP, while others consume these energy carriers for assimilation of C, N, and S to produce biomass (for review: Scheibe and Dietz 2012). Chloroplasts are the major sites of origin of reducing equivalents and ATP required for assimilatory processes. But on the other hand, chloroplasts and mitochondria are also the source of radicals. Any reactive forms of oxygen and nitrogen (ROS/RNS) resulting from over-reduced electron transport chains are scavenged by multiple antioxidant systems. Since radicals are inevitably formed during metabolic activities, antioxidant systems are present at sufficiently high levels in all compartments which can be enhanced when required (Foyer and Noctor 2011). On the other hand, the early increase of ROS is an important signal to induce systems for defense and repair (Mittler 2016). However, adaptation can also initiate reshuffling of the incoming energy into productive pathways for assimilatory processes. Therefore, it seems to be important to consider the actual conditions in a cell for analysis of productivity and stress responses. Time course and intensity of the applied challenge together with nutrient availability determine whether positive effect or damage is the result of a change of conditions. Sites of energy conversion and distribution as well as the regulatory principles acting in successful adaptive responses are discussed in this review. The Calvin–Benson cycle (CBC), the malate valves, the alternative oxidases, and major steps of reductant generation from the OPP pathway, triose-P oxidation, as well as glycolysis are described as examples for energy fluxes. Components of these major metabolic pathways are tightly linked to sensing of imbalances and initiating responses.

## Short-term adaptation to incoming light intensities and protection from oxidative stress

ROS generation is intimately interlinked with cellular redox-processes in photosynthesis and respiration but does not lead to biomass production. With an excess of incoming energy, the danger of oxidative stress is even increasing. In order to decrease the negative effects, many mechanisms exist in plants and in algae to allow for short-term responses of the electron flow in the thylakoids when coping with fluctuating input of light. The increase of ROS levels resulting from most types of impact leads to induction of antioxidant activities as part of the general adaptation syndrome first described in medicine for human stress (Selye 1950). Antioxidant enzymes are an essential part of the defense response and do not contribute to biomass production but rather to biomass consumption. As markers that indicate a response to oxidative stress, expression levels of 2-Cys peroxiredoxin (Prx), superoxide dismutase (SOD), and ascorbate peroxidase (APX) or the transcriptional repressor ANAC089 are usually monitored (Pulido et al. 2010; Dietz and Pfannschmidt 2011; Oelze et al. 2012; Klein et al. 2012). Ascorbate and glutathione are generally sufficient as redox buffers for most physiological requirements when changing conditions as fluctuating light or shift of the nutritional status disturb the cellular redox state (Foyer and Noctor 2011). Photoprotection at the cellular level is realized in many ways, starting with the closure of photosystems to decrease the amount of absorbed and excited energy and dissipation of the absorbed energy by a number of mechanisms, e.g., the xanthophyll cycle, state transitions, and contributions of various proteins to cyclic electron flow (Ruban et al. 2012; Hanke and Scheibe 2018; Alric and Johnson 2017). In particular, in lower photosynthetic organisms such as Chlamydomonas or Marchantia, and in cyanobacteria, the contributions from PGR5, PGR5-like, and flavodiiron proteins appear to be essential in particular under fluctuating light conditions (Alric 2010; Allahverdiyeva et al. 2013; Steinbeck et al. 2015; Shimakawa et al. 2017; Jokel et al. 2018). Faster recovery from the protected states in fluctuating light was suggested to help improve yield (Kromdijk et al. 2016). The repair machinery for the D1 protein prevents damage at this crucial point after energy capture in the reactive center of photosystem II (Theis and Schroda 2016). If the environmental change, however, is as substantial as to overstress the cell’s defense and repair mechanisms, an increase of ROS will be detectable as a result of “oxidative stress,” finally leading to cell death and necrosis.

When light-generated reductants are not used, the removal of energized electrons via water–water cycles (Beck/Halliwell/Asada pathway or NTRC/peroxiredoxin), photorespiration, or alternative oxidase (AOX) avoids radical formation in the electron transport chains both in chloroplasts and mitochondria (Scheibe et al. 2005; Sunil et al. 2013; Voss et al. 2013). The antioxidant systems need NADPH for regeneration provided by linear electron flow. Through the OPP pathways, both in plastids and cytosol, an alternative source for NADPH by carbohydrate oxidation is available in plants when required during dark metabolism. An electron acceptor limitation at PSI due to the lack of the major ferredoxin (Fd2) in a knockout line of Arabidopsis leads to insufficient rates of reductant generation and even results in oxidative activation of the plastidial G6PDH in the light (Voss et al. 2008). In mitochondria, NADP-isocitrate dehydrogenase provides reductant for the NTR system (Møller 2001).

## Rapid flux adjustments by post-translational regulation of chloroplast enzymes

Various steps in the Calvin–Benson cycle (CBC) are controlled by light/dark-modulated enzymes (Buchanan and Balmer 2005) to maintain homeostasis even under changing conditions. For reversible mediation of redox modifications to the various target proteins, a large number of thioredoxins and glutaredoxins are present in each compartment (Meyer et al. 2008). Metabolism determines the actual flux through the respective step by changing the rates of the redox interconversions between reduced and oxidized forms of these enzymes individually (Scheibe 1991). On the one hand, this mechanism allows for diurnally separated fluxes through reductive and oxidative pentose-phosphate cycle, respectively. But most importantly, continuous adjustment of enzyme activities is possible during illumination (Scheibe 1991; Knuesting and Scheibe 2018). As a key step of the CBC, the heterotetrameric isoform of GAPDH (GapA/B) is redox- and metabolite-controlled in its light/dark modulation (Baalmann et al. 1995, 1996). Both, reduced thioredoxin and the substrate 1,3bisPGA, determine the active portion of the enzyme required for the actual flux. As another example, the stromal FBPase activity is strictly following the demand as communicated by rising stromal concentrations of the substrate FBP. In addition to its role as a substrate, FBP functions as a positive effector for reductive activation of FBPase, and as the inhibitor of oxidative inactivation, with both effects acting on the redox-cycle resulting in the required enzyme activity at any time at this step of the CBC (Scheibe 1991). On the other hand, the lack of electron acceptor and an increased NADPH level (i.e., a low concentration of the inhibitor NADP^+^) causes the malate valve to open via reductive activation of the NADP-dependent malate dehydrogenase (NADP-MDH) for indirect export of NADPH in illuminated chloroplasts. In contrast, if reducing equivalents are consumed for 3-PGA reduction, the higher NADP^+^/[NADPH + NADP^+^] ratio inhibits NADP-MDH activation and prevents export of reducing equivalents from the chloroplasts (Scheibe 2004).

Such autoregulatory mechanism is making use of substrates or products of an enzyme for fine-tuning its activity specifically, thus supporting homeostasis of metabolism even under changing conditions (Faske et al. 1995; Holtgrefe et al. 1997). The hierarchy of the various electron acceptors is evident in experiments with isolated chloroplasts under defined metabolic conditions (Backhausen et al. 1994, 2000). CO_2_ assimilation as the major reductive pathway is continuously fed with energy, but any sulfate or nitrite will be reduced directly with Fd_red_ prior to NADPH generation. Enzyme regulation allows for adaptation of the plant to cope with an increased energy input and usage of electrons preferentially for biomass production. An increase of the light intensity immediately leads to some over-reduction of the components of the electron transport chain, and the system responds with oscillations that are dampened due to the changed enzyme activities to reach a new steady state. The rapid activation of NADP-MDH and a subsequent decrease of activity when the redox balance has been re-established prevents any severe imbalances (Scheibe and Stitt 1988). Similar responses adjust the fluxes through the various parts of the CBC. Such rapid responses, however, are only possible due to the post-translational modification of enzymes that are already present. Increased enzyme levels can only be achieved by gene transcription and translation requiring more time (see “Moonlighting and multitasking of enzymes involved in energy metabolism” section).

## Distribution of assimilates and energy across compartment borders

**Fig. 1:**
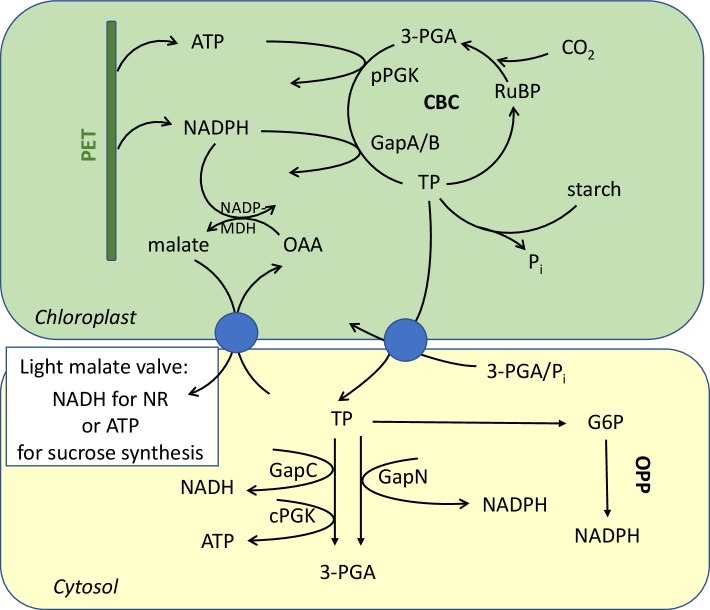
Generation and indirect transport of reducing equivalents and ATP. Excess NADPH from the light reactions, not needed for stromal metabolism, e.g., CBC, or for balancing the ATP/NADPH ratio, is transformed to malate by the light/dark-modulated NADP-dependent malate dehydrogenase (NADP-MDH). Malate can be used to generate NADH in the cytosol or ATP in the mitochondria. When NADPH is needed as a reductant in the cytosol, either the irreversible GapN (non-phosphorylating GAPDH) or the oxidative pentose-phosphate (OPP) pathway oxidize assimilates (TP or G6P) for the sake of protection from oxidative stress, defense, and repair

The primary products of carbon assimilation are the triose phosphates (TP). Five out of six molecules produced during three CBC turnovers are used for the regeneration of the CO_2_ acceptor ribulose 1,5-bisphosphate (RuBP), while every sixth one is a net product and can be partitioned either into transitory starch or into sucrose synthesis in the cytosol after TP export. The TP-phosphate translocator (TPT) functions as an antiporter, shuttling TP in exchange with inorganic phosphate (P_i_) or 3-phosphoglycerate (3-PGA), the direction depending on the actual metabolic fluxes (Flügge and Heldt 1984) (Fig. 1). Export of TP can be linked to the indirect export of NADPH and ATP, and, therefore, it can also serve as another means for shuffling energy across membranes for the supply of neighboring compartments. The release of the indirectly transported energy can occur in the glycolytic steps catalyzed by cytosolic NAD-GAPDH (in plants GapC) and 3-phosphoglycerate kinase (PGK) to provide NADH and ATP for cytosolic metabolism. Alternatively, a plant-specific glyceraldehyde 3-phosphate dehydrogenase (GapN) catalyzes an irreversible oxidation of the aldehyde (TP) to yield the acid 3-PGA without coupling the oxidation with substrate phosphorylation and ATP generation **(**Fig. 1). Such indirect export of reductant from the chloroplast appears to be used when neither ATP nor NADPH are consumed in plastidial metabolism, e.g., due to the lack of CO_2_ during drought stress (Bustos et al. 2008). As expected, the lack of GapN in knockout plants leads to an increased expression of the cytosolic G6PDH isoforms 5 and 6 (Rius et al. 2006). In plants, NADPH can thus be formed from oxidation of glucose-6-P (G6P) or triose-P (TP), respectively, by cytosolic glucose 6-phosphate dehydrogenase (G6PDH) and, specifically in plants, by non-phosphorylating GAPDH (GapN). GapN has been shown to be rather stable under oxidizing conditions (Piattoni et al. 2013). An increased G6PDH or GapN activity, therefore, enables maintenance of redox homeostasis before any oxidative damage would occur during short-term stress (Landi et al. 2016).

Although cytosolic enzymes are potential sources of NADH and ATP (glycolytic step catalyzed by GapC/PGK, and by NAD-malic enzyme), provision of NADH for nitrate reduction and of ATP for sucrose synthesis through interorganellar transport originating from chloroplasts and mitochondria, respectively, has been shown to be necessary for net production (Gardeström and Igamberdiev 2016; Scheibe 2004; Krömer and Heldt 1991). As described in “Rapid flux adjustments by post-translational regulation of chloroplast enzymes” section, the chloroplast NADP-MDH as part of the malate valve operating in the light for export of excess NADPH is strictly controlled by the NADP^+^-to-NADPH ratio acting on the redox-cycle between reduced and oxidized enzyme form driven by the ferredoxin-thioredoxin system and the concomitant reoxidation of the enzyme. It allows for indirect export of reducing equivalents only when they are in excess (Scheibe 2004). Malate can then be used in many ways in the various compartments that possess MDH activities of the NAD-dependent isoforms. The relevant membranes are equipped with the specific dicarboxylate transporters that operate along the concentration gradient determined by production and consumption on each (Selinski and Scheibe 2018).

A different type of malate valve to maintain redox balance in plastids of non-green cells or in chloroplasts in the absence of light could be identified. NADH generated in the glycolytic step by the plastidial NAD-GAPDH (GapCp in *A. thaliana*) will be converted by a plastidial NAD-MDH resulting in the so-called dark malate valve (Backhausen et al. 1998b; Scheibe 2004) (Fig. 1). For metabolic processes, therefore, NADPH (from glucose 6-P (G6P) in the plastidial oxidative pentose-phosphate (OPP) pathway) and ATP (from substrate phosphorylation in plastidial glycolysis) are generated independently at the required rates.

The interplay between light reactions and mitochondrial metabolism has been suggested to play an important role to optimize photosynthesis (Raghavendra and Padmasree 2003). The particular role of AOX in avoidance of ROS formation under conditions such as high light or drought (lack of acceptor CO_2_) has been demonstrated in transgenic lines lacking the major isoform of the alternative oxidase AOX1A (Strodtkötter et al. 2009). AOX1A is required for the controlled release of unused electrons under high light intensities. An increased expression of AOX1D does not entirely alleviate the lack of AOX1A in the mutant plants. The specific fine-tuning of the activity of each AOX isoform appears to be achieved at multiple levels, including the post-translational modifications of the reduced (activated) proteins by specific tricarboxylic acid cycle intermediates (Selinski et al. 2017, 2018a). Again, metabolites determine the activities of a valve to release any excess reducing power that has not been used elsewhere in the cell as heat only when indicated by changing metabolite pools (Selinski et al. 2018b). In this sense, AOX activities are determined by redox state and mitochondrial metabolism, and they act as a sensor of imbalances due to the regulatory properties of this valve functioning for the final non-destructive dissipation of excess electrons as thermal energy.

## Energy distribution according to kind and availability of nitrogen source

**Fig. 2:**
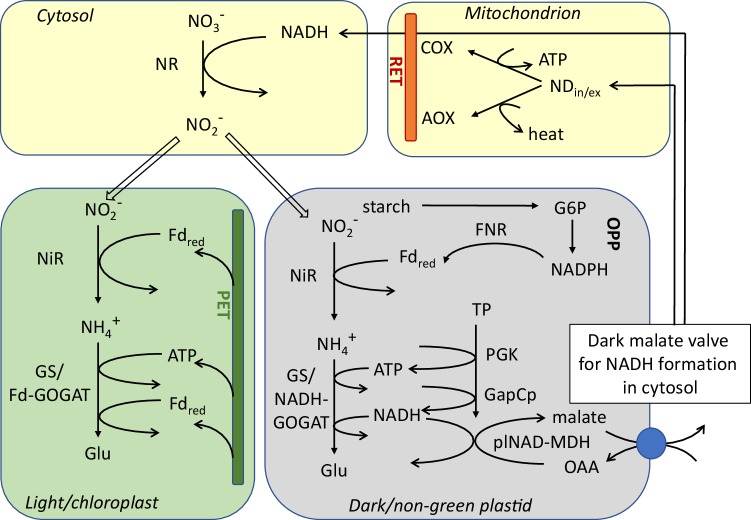
Energy requirements for N-assimilation as dependent on the N-source. Nitrate and ammonium assimilation require different kinds of reductant and ATP in the various compartments. Balancing of the ATP/NAD(P)H ratio and indirect transport of the energy carriers as necessary in light and dark is achieved by the provision of reduced ferredoxin (Fd_red_), NADPH, ATP, and NADH for the respective isoenzymes. In darkened chloroplasts or non-green plastids, ATP and Fd_red_ are obtained via plastidial glycolysis at the substrate phosphorylation step of plastidial NAD-GAPDH (GapCp) in conjunction with the plastidial OPP pathway and the dark malate valve

Not only the availability of light and CO_2_, but also the type of N-source, whether nitrate or ammonium as a nutrient, is a challenge for plants. Although nitrate is the preferred nitrogen source, ammonium can also be assimilated since the required enzymes are present due to the photorespiratory NH_4_^+^ re-assimilation in all C3-plants. N-assimilation can take place either in green tissues or directly in root cells (Fig. 2). Photorespiration itself with its energy-consuming glycollate detoxification pathway provides another option for buffering and rebalancing the energy status during stress (Wingler et al. 2000; Hodges et al. 2016).

Integration and cooperation of these metabolic activities pose some problems, since energy carriers required in the various cellular compartments need to be shuttled indirectly across membranes (Scheibe 2004; Taniguchi and Miyake 2012). In the absence of photosynthesis, electrons are made available by carbohydrate oxidation (glycolysis or OPP pathway) (Hansen et al. 1998). Therefore, the aspects of macro- and micro-compartmentation need to be taken into account as already mentioned in the context of CO_2_ assimilation. Metabolite transporters, as well as the formation of metabolons, facilitate such complex metabolic networks (Sweetlove and Fernie 2013).

The requirement for reductants and ATP differs considerably, also as the sites of usage are concerned, when short-term or long-term exclusive ammonium supply needs to be coped with (Fig. 2). Short ammonium exposure (3 h) of Arabidopsis plants grown originally on nitrate resulted in a fast response to achieve protection from oxidative stress (Podgórska et al. 2017), with increases in antioxidative enzymes, such as CuZn-SOD, and induction of alternative electron transport pathways in mitochondria (Escobar et al. 2006). Under these conditions, less reductant is needed and is dissipated via AOX to prevent radical formation. Upon a long-term exposure, ammonium causes adverse effects on the cellular redox balance and the adenylation status, mainly in the extrachloroplastic fraction, and on mitochondrial ROS production, resulting in massive growth retardation, but chloroplasts appeared to remain functional (Podgórska et al. 2013). As an increased NADH-GOGAT is necessary for successful NH_4_^+^ assimilation (Konishi et al. 2014), Arabidopsis plants with a decreased expression of the plastidial NAD-dependent MDH (plNAD-MDH) could survive growth on ammonium better than the wild type, due to a compensatory increase of NADH-GOGAT (Selinski and Scheibe 2014). Interestingly, plants lacking the complex I in the respiratory electron transport chain in mitochondria (*frostbite1*) also exhibited improved growth on ammonium compared to nitrate (Podgórska et al. 2015).

Under nitrate nutrition, requirements for reductant and ATP supply differ entirely from ammonium conditions (Fig. 2) (Escobar et al. 2006). In particular, large amounts of electrons are required in the plastids for nitrite reduction. In green cells, provision of plastidial reductants results from photosynthetic electron flow taking electrons from reduced ferredoxin (Fd_red_) directly. In non-photosynthetic conditions, NADPH generated in the plastidial OPP pathway together with ferredoxin-NADP oxidoreductase (FNR) to reduce the root ferredoxins is required. Indeed, expression levels of the plastidial isoforms of glucose 6-phosphate dehydrogenase (G6PDH2 and 3) are increased in roots and shoots of nitrate-grown plants (Wang et al. 2003; Bussell et al. 2013). Knockout plants for NADP-MDH compensate the lack of the malate valve with increased expression of alternative systems for reductant dissipation but exhibit improved growth on nitrate as N-source when compared to wild type (Hebbelmann et al. 2012). Taken together, the lack of malate valves for export of NADH and of NADPH from plastids in darkness or during illumination, respectively, leads to a shift in reductant availability that improves either NH_4_^+^ or NO_3_^−^ assimilation compared to wild type (Selinski and Scheibe 2014).

## Moonlighting and multitasking of enzymes involved in energy metabolism

A complex network of reactions during primary metabolism is characterized by multiple control points for flexible integration and adjustment of fluxes dependent on supply and demand in accordance with changing environmental conditions. Sustained environmental stress factors, e.g., shortage of nutrients and the presence of abiotic stressors affecting developmental programs, are perceived and responded to through altered gene expression when the capacities of the regulated enzymes are exhausted. Input signals (redox state and metabolite levels) generated from a change of conditions or the incidence of different types of stress are integrated to yield very specific answers. Responses at all levels of regulation and over the total time span after application of an environmental change such as increase of light intensity become evident (Dietz 2015).

The glycolytic enzyme GAPDH (GapC1 and 2 in *A. thaliana*), as well as aldolase, is subject to modulation by the redox status of the cytosol, namely by S-glutathionylation and S-nitrosylation of their cysteine residues, resulting in reversible or irreversible inactivation, depending upon the presence of substrate that prevents the inactivation (Holtgrefe et al. 2008; van der Linde et al. 2011). A role of glycolytic enzymes such as GAPDH has been suggested already as a redox sensor for H_2_O_2_ increase (Hancock et al. 2006). There is now a growing number of publications describing the “moonlighting” properties of enzymes involved in central energy metabolism, namely the cytosolic glyceraldehyde-3-P dehydrogenase (NAD-GAPDH, GapC in plants), in all organisms (Sirover 2011; Hildebrandt et al. 2015). Redox-dependent association of GAPDH with mitochondria was suggested to induce improved energy generation. We found these glycolytic enzymes associated with the actin cytoskeleton and with mitochondria attached via the voltage-dependent anion channel (VDAC) (Wojtera-Kwiczor et al. 2012; Schneider et al. 2018). The redox-dependent change of the properties of the modified protein is the basis for changes in activity, subcellular localization, and protein–protein interactions in the context of many more cellular responses yet to be identified. As a target of H_2_O_2_, cytosolic GAPDH has been suggested to mediate any redox imbalance in a signaling cascade to induce antioxidant defense (Hancock et al. 2005). The high sensitivity of the catalytic cysteine residue present in the active site of GAPDH towards oxidation provides the basis for its prominent role as a central regulator (Peralta et al. 2015; Zaffagnini et al. 2016). There are some evidences that redox-imbalances trigger the nuclear localization of GapC (Schneider et al. 2018). As a well-studied example in the mammalian system, an association of S-nitrosylated GAPDH with SIAH1, an E3-ubiquitin ligase, leads to nuclear translocation and induction of cell death (Hara et al. 2005). In fact, a similar protein, namely SINAL7, was identified as a binding partner of GAPDH in plants (Peralta et al. 2016), suggesting moonlighting functions of the metabolic enzymes in plants as also evidenced in yeast and animals. Furthermore, cytosolic GAPDH was detected in nuclei of cadmium-treated roots (Vescovi et al. 2013).

With the aim to identify cis-elements and proteins of the transcriptional machinery involved in the induction of NADP-MDH expression as seen under high light in short-day-grown plants (Becker et al. 2006), we have performed a yeast-one-hybrid screen with gene fragments of the NADP-MDH gene. Interestingly, the glycolytic enzymes GapC and aldolase were identified as prominent binding partners of these fragments comprising parts of the coding sequence and an intron (Hameister et al. 2007). We could show that under conditions of excessive illumination, NADP-MDH transcript is increased, but only in actively metabolizing cells in leaves of vegetatively growing plants under short-day photoperiod (8 h light, 16 h dark) (Becker et al. 2006). In contrast, plants grown under long-day photoperiod that are flowering-induced and aim for finishing their life cycle as soon as possible with seed production induce only protective mechanisms for ROS scavenging, but not NADP-MDH expression. Consequently, the highest capacities of NADP-MDH were found in growing leaves of young tobacco plants (Faske et al. 1997; Backhausen and Scheibe 1999). The assumption that NADP-MDH contributes to improved growth was also confirmed when potato plants that overexpress NADP-MDH were found to grow faster than antisense plants when they were kept under ambient (e.g., fluctuating light) conditions (Backhausen et al. 1998a). Imbalances in the photosynthetic electron transport chain lead to a signal transfer via oxidation of cytosolic GAPDH and its nuclear translocation to activate transcription of NADP-MDH (Zachgo et al. 2013; Hildebrandt et al. 2015). When grown in long-day photoperiod (as is usually the case when rapid growth and reproduction are the primary aims of experimental plant cultivation), protection from oxidative stress or cell death is induced upon environmental challenges, and no further acclimation or improvement of metabolism is observed.

The various isoforms of MDH present in all compartments together with dicarboxylate translocators of the organellar membranes allow for interorganellar communication (Selinski and Scheibe 2018). In this respect, a central role of peroxisomal NAD-MDH is suggested for interorganellar communication in Chlamydomonas (Kong et al. 2018). Interestingly, as is known for GapC, cytosolic NAD-MDH1 appears to be subject to redox modifications as well (Hara et al. 2006), and the reversible oxidation of its C-terminal cysteine residue might help to protect the enzyme from oxidative damage (Huang et al. 2017). Redox-dependent signaling and effects on gene expression might, therefore, also derive from imbalances between the compartments, sensed by the involved enzymes and transferred to the nucleus. Upon redox-imbalances and ROS formation due to stress impact, plants can adapt and acclimate to various stress factors resulting even in cross-tolerance (Locato et al. 2018). Malate itself has now been suggested to serve as a signal in cases of imbalances between chloroplast and mitochondria (Zhao et al. 2018). Malate as a signal and the nucleo-cytosolic occurrence of NAD-MDH are most likely indicators for redox-imbalances not only in plants, but also in mammalian cells when adaptation to metabolic imbalances is required and p53 transcriptional activity is induced upon metabolic stress (Lee et al. 2009).

## Redox-regulation at all levels for optimal adaption of energy metabolism

In addition to causing oxidative stress and damage, ROS/RNS can cause oxidative cysteine modifications such as the reversible formation of disulfide bridges, S-glutathionylation, and S-nitrosylation found to occur at various target proteins. These post-translational redox modifications are components of regulatory systems on the one hand controlling enzyme activities diurnally as on/off switches, and during illumination by fine-tuning of light–dark modulation of chloroplast enzymes (Scheibe 1991; Knuesting and Scheibe 2018). On the other hand, when cytosolic enzymes of central energy metabolism are affected upon a shift towards oxidizing conditions, they act in signal transduction pathways involved in transcriptional regulation (Zachgo et al. 2013; Hildebrandt et al. 2015). For such cases of regulation, ROS are good (Mittler 2016).

Light–dark modulation and fine-tuning by metabolites in the case of the thioredoxin-dependent of reversible redox modifications of preexisting chloroplast enzymes allows for fast responses to changing conditions, but only within the range of activity that is limited by its presently available full capacity (100% of its activity). However, sustained demand for the full capacity results in induction of gene expression and the increased synthesis of the limiting enzyme. Since the respective chloroplast enzymes are nuclear encoded, retrograde signaling from the chloroplast to the nucleus has to be assumed. This scenario requires a signal to be transferred through the cytosol. Since apparently, post-translational redox modifications affecting activities are not only observed in chloroplasts but also in other cellular compartments, such signal can be transduced to the cytosol. Concerning the glycolytic enzymes in the cytosol, not only their activities but also their subcellular localizations and binding properties are modified allowing for their moonlighting functions. Enzymes of central energy metabolisms such as GapC or cytosolic MDH act as sensors to integrate incoming information on the actual energy status. The output allows for adjustment of metabolic fluxes to variable conditions. Therefore, GapC and cytosolic MDH are central not only as part of the energy fluxes but also as hubs to link redox state and energy-requiring metabolism in this highly complex network. Oxidative inactivation by modification of sensitive cysteine residues can elicit changes in energy metabolism depending on the general situation of the cell. At both ends, namely in chloroplasts and in mitochondria, malate valve and AOX, respectively, respond directly to and can also be seen as sensors of energy status for maintenance of homeostasis.

## Conclusions and outlook

Plants possess the capability to avoid imbalances or any significant increase in ROS. In most situations which are not destructive, cellular homeostasis is maintained over a wide range of conditions. Under optimal conditions in nature, and in carefully controlled experimental setups, the changed protein–protein interactions and localizations of GapC causative for induction of the respective genes result in sustained or even increased biomass production and avoidance of oxidative stress. A sudden, massive change of light intensity, however, leading to a more pronounced and eventually toxic increase of ROS, induces transcription of defense genes, or programmed cell death, or leads to uncontrolled damage and necrosis. The signal transduction networks of plants are highly complex due to their ability to integrate multiple kinds of information for a proper response. In this challenging situation, metabolism and redox homeostasis permanently require adjustment and optimization (Kocsy et al. 2013; Mittler 2016; Foyer et al. 2017a). Cytosolic GAPDH and the isoforms of MDH and AOX as control hubs during active metabolism are capable to sense and mediate incoming challenges.

The role of GapC in improving biomass production by redirecting energy fluxes according to the light and nutrient availability requires analysis of mechanisms at multiple regulatory levels. To improve productivity by increasing the efficiency of photosynthesis, the aspect of adaptation and the required signals and time span to realize an altered machinery for flexible responses need to be taken into consideration (Foyer et al. 2017b; Bailey-Serres et al. 2018). It is crucial to obtain a better understanding of the interaction of the various mechanisms that help to fine-tune the responses of plant cells to changing environments, when aiming for “better plants” to be created by biotechnological approaches (Kramer and Evans 2011; Kerchev et al. 2015). Any breeding or biotechnological approaches aiming for a productive outcome should have in mind the complex regulatory network to avoid failure of seemingly straight-forward approaches. The actual situation in a cell or plant is determined by its redox state (normal or stressed) and its metabolic status (fasting and feeding). Failure of the coordination of nutritional status with growth activities is detrimental to the success in biomass production (Dietz et al. 2010; Dolferus 2014).

## Abbreviations

AOX: Alternative oxidase
ADP: Adenosine diphosphate
ATP: Adenosine triphosphate
COX: Cytochrome *c* oxidase
CBC: Calvin–Benson cycle
Fd_red_: Ferredoxin (reduced)
GAPDH: Glyceraldehyde 3-phosphate dehydrogenase
Glu: Glutamate
G6P: Glucose 6-phosphate
G6PDH: Glucose 6-phosphate dehydrogenase
MDH: Malate dehydrogenase
NAD(P)(H): Nicotinamide adenine dinucleotide (phosphate) (reduced)
ND_in/ex_: NADH dehydrogenases (internal and external)
NR: Nitrate reductase
NiR: Nitrite reductase
OAA: Oxaloacetate
OPP: Oxidative pentose-phosphate
PGA: 3-phosphoglycerate
PGK: 3-phosphoglycerate kinase
PET: Photosynthetic electron transport
RET: Respiratory electron transport
RuBP: Ribulose 1,5-bisphosphate
TP: Triose phosphate
TPT: Triose-phosphate-phosphate translocator
VDAC: Voltage-dependent anion channel
